# All-Cause Excess Mortality During the COVID-19 Pandemic in a Rural Area of North India

**DOI:** 10.7759/cureus.116525

**Published:** 2026-09-19

**Authors:** Mahesh Satija, Anurag Chaudhary, Pranjl Sharma, Surinder P Singh, Nishant Sharma, Sarit Sharma, Sangeeta Girdhar, Vikram K Gupta, Priya Bansal

**Affiliations:** 1 Community Medicine, Dayanand Medical College and Hospital, Ludhiana, IND; 2 Biostatistics, Community Medicine, Dayanand Medical College and Hospital, Ludhiana, IND

**Keywords:** covid-19, excess mortality, pandemic, punjab, rural

## Abstract

Introduction

COVID-19 pandemic significantly impacted global mortality; however, many deaths were underreported. Excess mortality explains both direct and indirect impacts of the COVID-19 pandemic and therefore provides true and better estimates of pandemic deaths.

Objectives

The objective of this study was to estimate excess all-cause mortality during the COVID-19 pandemic in a rural area of North India.

Methods

The study was carried out in the field practice area of the Rural Health Training Centre (RHTC) of a tertiary care hospital in North India. This area covers fifteen villages where home-based comprehensive health care is provided. Age, gender, and month-wise mortality data of these villages (for the years 2015-2023) were extracted from the records. Two models, historical five-year average and negative binomial regression, were used to estimate all-cause excess mortality for the COVID-19 period (January 2020-May 2023) compared with pre-pandemic years (2015-2019). Excess mortality was estimated as the difference between the number of all-cause deaths and baseline expected deaths for each model. Percentage excess mortality (ratio of excess deaths to expected deaths) was calculated.

Results

Monthly all-cause excess mortality was observed to be higher (exceeding 95% CI upper bounds) during the months of April, May, and August 2021 than expected mortality. May 2021 had the highest excess mortality, consisting of 47 and 43 deaths (223.8%, 167.4%) using historical average and negative binomial regression analysis, respectively. However, it was lower than expected in 2020, 2022, and 2023. Males and adults aged 40-59 years had higher excess mortality. Overall excess mortality was 19 (1.8%), with the year 2021 reporting high excess mortality of 72 (24%).

Conclusions

Findings indicate significant excess all-cause mortality during the COVID-19 pandemic. The study witnessed high excess mortality across age groups, particularly among 40- to 59-year-old adults and males.

## Introduction

The global coronavirus (COVID-19) pandemic significantly affected human lives and is going to be remembered for generations. World Health Organization (WHO) declared COVID-19 a Public Health Emergency of International Concern (PHEIC) on 30th Jan 2020 [1]. Globally, since the beginning of the pandemic in December 2019, around 780 million COVID-19 cases and over 7.1 million deaths were reported [2]. An accurate estimate of the number of deaths is vital to understand the complete public health impact of the COVID-19 pandemic. However, the actual number of COVID-19 deaths is believed to be higher than the reported number and may not reflect the true burden of COVID-19 mortality [3].

With an initial 171 COVID-19 deaths in January 2020, it increased sharply to over 18 lakh deaths by the end of the year. However, preliminary WHO estimates suggested at least three million deaths attributable to COVID-19 in 2020, which is about 1.2 million higher than officially reported mortality [1]. Globally, various factors led to underestimation of COVID-19 mortality. Many deaths would not have been reported due to the non-availability of COVID-19 testing, especially during the initial phase of the pandemic; variations in the definition of COVID-19 deaths as the underlying cause of death; many patients avoiding utilization of healthcare facilities for COVID-19 care; the effect of lockdown; and political considerations in various places [3,4].

Monitoring of COVID-19 mortality was vital to track how the pandemic evolved over time. However, timely and accurate data on vital statistics, including causes of death, are still not available in many countries due to non-functioning civil registration systems. Death registration ranged from 10% in the African region to 98% in the European region in an estimate from 133 countries [1]. 

Measurement of excess mortality from all causes is a more objective method and provides standardized estimates of COVID-19 deaths. Excess mortality explains both the direct and indirect impacts of the COVID-19 pandemic and therefore provides true and better estimates of COVID-19 deaths. Excess mortality is defined as the difference between the reported all-cause deaths and the expected number of deaths based on past trends in all-cause mortality in a particular place and time period in the absence of a pandemic. Many countries have used various processes for testing and reporting of COVID deaths, therefore making comparisons difficult. To overcome these challenges, many countries have turned to excess mortality as a more accurate measure of the true impact of the pandemic [3,4].

Prior attempts estimated that globally there were 18.2 million excess deaths during January 2020-December 2021, and South Asia, the Middle East, North Africa, and Eastern Europe regions reported higher excess mortality [4]. WHO estimates for the same period reported 14.83 million excess deaths globally, 2.7 times higher than the reported COVID mortality. Higher excess mortality was reported in males (57%) as compared to females (43%) and in older adults [5]. WHO declared the pandemic to be over as a public health emergency of international concern (PHEIC) on 5th May, 2023 [6]. India reported the first confirmed case of COVID-19 in Kerala on 30 Jan, 2020 [7]. There were around 45 million cases and over five lakh deaths reported due to COVID-19 till 5th May, 2023 [8]. Over four million COVID-related excess deaths were estimated during the pandemic (2020-21) in India [4,5], about eight times higher than those reported [4].

A report by the Government of Punjab, India, based on registered deaths under the Civil Registration System (CRS) between March 2020 and June 2021, suggested over 52,000 excess deaths in Punjab due to COVID, about 2.8 times higher than the reported COVID deaths [9]. Preliminary analysis on excess mortality due to COVID-19 revealed that there was a 120.3 excess mortality rate (per 100,000 population) as on 31st August, 2021 in Punjab [10]. However, another analysis estimated 135,000 deaths (Jan 2020-Dec 2021), registering an excess mortality rate of 227.1 [4]. Limited information is available about excess deaths due to COVID-19 in the state of Punjab, India, especially from rural areas. Therefore, the present study is an attempt to estimate excess all-cause mortality during the COVID-19 pandemic in a rural area of Ludhiana, Punjab, India.

## Materials and methods

This community-based cross-sectional study was carried out in the Rural Health Training Centre (RHTC), a rural field practice area in village Pohir, Block Dehlon, District Ludhiana, Punjab, India. It caters to 15 villages (30,706 population), where continuous home-based comprehensive health care is provided by the team of multipurpose health workers and village-level health workers and is regularly supervised by the faculty of the department. Information on demographic and health records of each family in these villages is maintained in family folders and regularly updated by the health workers. Village-level health workers are residents of that particular village in the field practice area, and hence even during the COVID pandemic, every death was recorded during the same time period/months as the deaths happened. Age, gender, and month-wise mortality data from the year 1st Jan 2015 to 31st December 2023 were extracted from these records of fifteen villages. Ethics approval was obtained from the Institutional Ethics Committee (DMCH/R&D/2021/89 dated 21.08.2021).

Statistical methods

Data were entered into Microsoft Excel (Microsoft, Redmond, Washington). WHO ended the COVID-19 pandemic emergency (PHEIC) on 5th May, 2023. Therefore, excess mortality for COVID was calculated for the event period covering 1st Jan 2015 to 31st May 2023. Expected number of deaths with 95% confidence interval limits (95% CI) for each month for Jan 2020- May 2023 were calculated using the historical five-year average and negative regression method from pre-pandemic years 2015-2019 [3,11].

To estimate the expected number of deaths during the pandemic period, a negative binomial generalized linear model was fitted using pre-pandemic mortality count data from 2015 to 2019. The model was specified as:

\[ ln(μᵢₜ) = β₀ + β₁t + γ(Ageᵢ) + δ(Sexᵢ) + Σ θₘ(Monthₘ) \]

In this formulation, ln(μᵢₜ) represents the natural logarithm of the expected number of deaths for stratum i at time t. The parameter β₀ denotes the model intercept, while β₁ captures the underlying secular time trend estimated from the pre-pandemic baseline period (2015-2019). The exponentiated coefficient (e^β₁) represents the multiplicative change in the expected number of deaths per unit increase in time. Categorical fixed effects for age group, γ(Ageᵢ), and sex, δ(Sexᵢ), were included to account for demographic differences in baseline mortality. Monthly indicator variables, Σ θₘ(Monthₘ), were included to account for recurrent seasonal variation. The fitted model was subsequently used to predict the expected number of deaths for the pandemic period from January 2020 through May 2023, representing the number of deaths expected in the absence of the pandemic based on the pre-pandemic mortality pattern. Excess mortality was estimated as the difference between the number of deaths from all causes during Jan 2020- May 2023 and expected deaths by historical average and negative regression models. Overall excess mortality with 95% uncertainty intervals (UI) was estimated. Percentage of excess mortality (ratio of excess deaths to expected deaths, in percentages) was also calculated.

Temporal validation was carried out with the following validation method/equation: training period - years 2015-2018; holdout - year 2019 (coverage = 98.89%). Similarly, sensitivity analysis was carried out comparing trend vs. no-trend baseline models. It demonstrated model robustness via a 2019 holdout temporal validation and sensitivity analysis to justify the overall parsimonious model. R software version 4.5.1 (R Core Team, R Foundation for Statistical Computing, Vienna, Austria), excess mortality calculator (shiny app) [11], and Microsoft Excel were used for performing analysis and generating graphs.

## Results

A total of 2637 deaths were recorded between 1st January 2015 and 31st December 2023. The crude death rate (number of total deaths per 1000 population) remained almost stable between 9.1 and 9.5 deaths per 1000 population per year during the pre-pandemic period (2015-2019). It increased sharply to 12.2 in 2021 during the COVID-19 pandemic and decreased to 9.3 and 9.4 deaths per 1000 population in 2022 and 2023, respectively (Figure 1).

**Figure 1:**
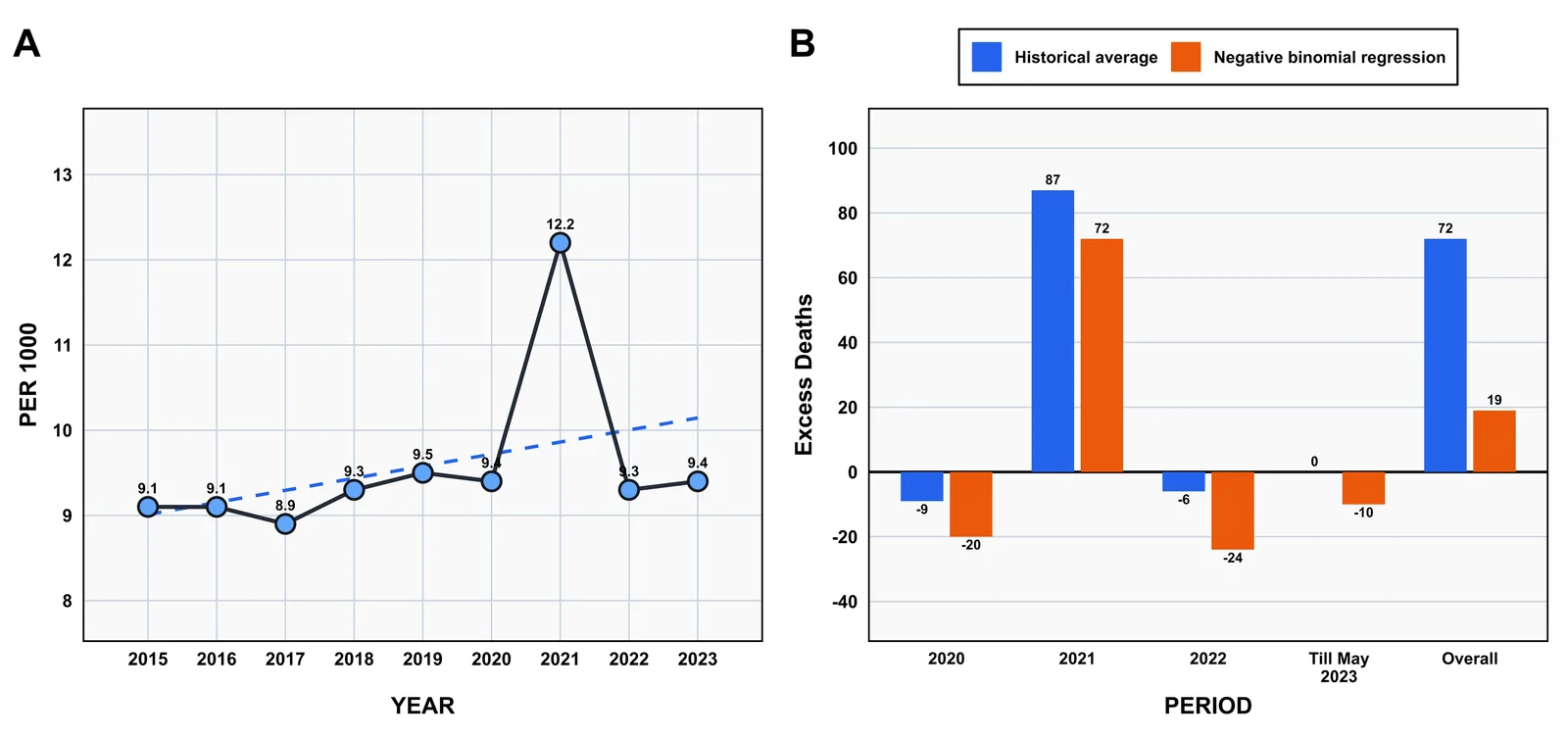
Crude death rate and excess mortality during the COVID-19 pandemic A) Crude death rate (Jan 2015-Dec 2023); B) Excess mortality during the COVID-19 pandemic period (Jan 2020-May 2023)

Monthly all-cause deaths surpassed the upper bounds (95% CI) of expected deaths in the months of April-May and August 2021 as per historical five-year average and negative regression models (Figure 2). The highest number of excess deaths (47, 43) and % excess mortality (223.8%, 167.4%) were observed in the month of May 2021 as per the historical average and negative binomial regression (NBR) model (Table 1). Excess mortality (72) and % excess mortality (23.9%) were higher in the year 2021, while years 2020, 2022 and 2023 reported lower deaths than expected levels (-20, -24 and -10, respectively) and % excess mortality (-6.7%, -7.9% and -7.8%, respectively) as per NBR analysis. Thus, from 2022 onwards, all-cause mortality returned to normal or baseline levels (Table 1).

**Figure 2:**
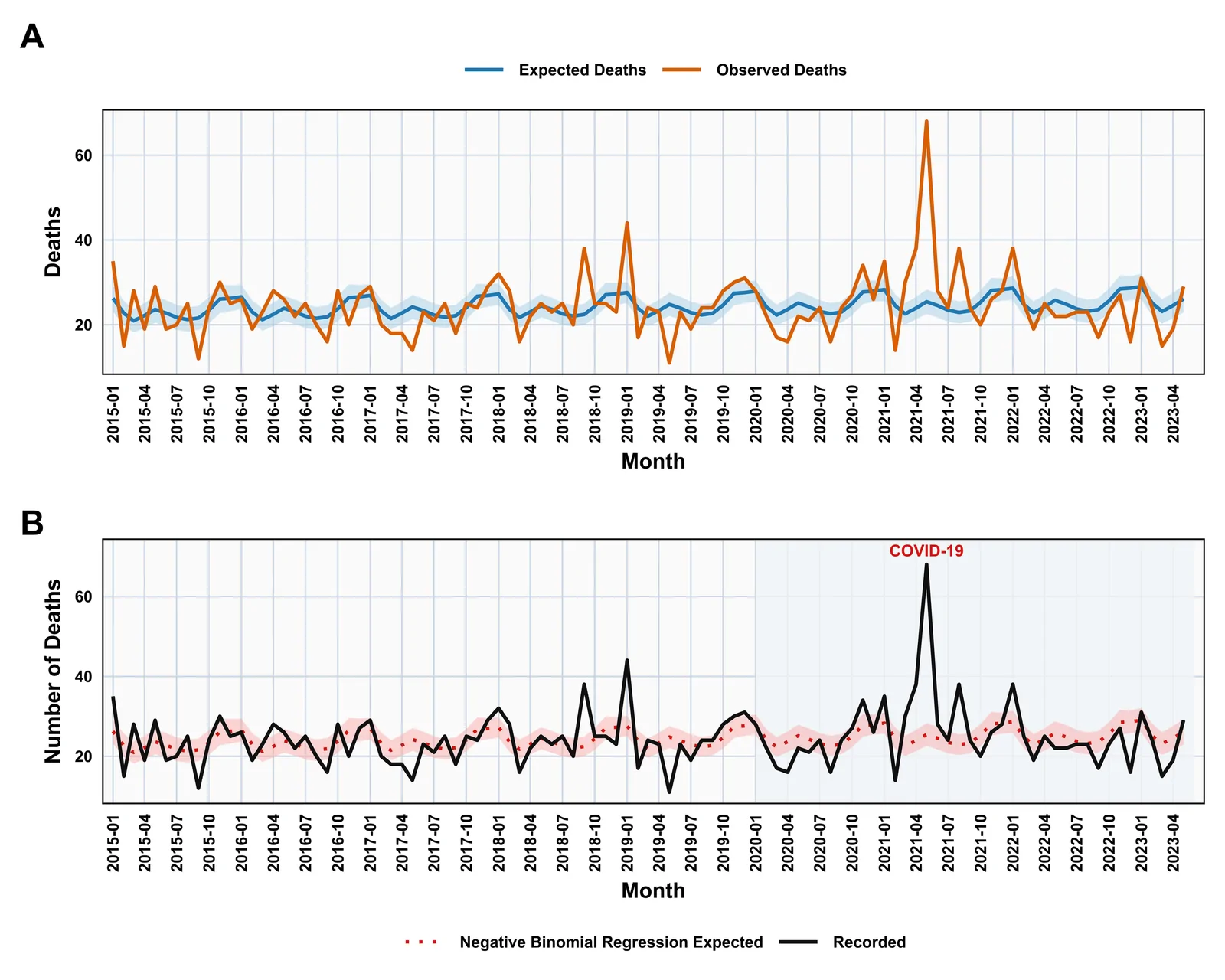
Monthly all-cause mortality (Jan 2015-May 2023) A) Historical average model; B) Negative binomial regression model

**Table 1:** Excess deaths through the COVID-19 pandemic by month and year (Jan 2020-May 2023)

Month/Year	2020		2021		2022		2023	
	Excess deaths N(%)		Excess deaths N(%)		Excess deaths N(%)		Excess deaths N(%)	
	5-year average	Negative binomial regression	5-year average	Negative binomial regression	5-year average	Negative binomial regression	5-year average	Negative binomial regression
January	-5 (-15.2)	0	2 (6.1)	7 (23.7)	5 (15.2)	9 (32.7)	-2 (-6.1)	2 (6.9)
February	2 (10)	-2 (-9)	-6 (-30)	-10 (-42.8)	5 (25)	0	4 (20)	-1 (-4.4)
March	-5 (-22.7)	-5 (-23.7)	8 (36.4)	7 (33)	-3 (-13.6)	-4 (-17)	-7 (-31.8)	-8 (35.2)
April	-6 (-27.3)	-8 (-32.3)	16 (72.7)	14 (59)	3 (13.6)	1 (3.2)	-3 (-13.6)	-6 (-22.5)
May	1 (4.8)	-3 (-12.4)	47 (223.8)	43 (167.4)	1 (4.8)	-4 (-14.6)	8 (38.1)	3 (11.2)
June	-1 (-4.5)	-3 (-13.4)	6 (27.3)	3 (14.1)	0	-3 (-11.5)	NA	NA
July	2 (9.1)	1 (3.7)	2 (9.1)	1 (2.4)	1 (4.5)	-1 (-3.1)		
August	-7 (-30.4)	-7 (-29.2)	15 (65.2)	15 (66)	0	0		
September	2 (9.1)	1 (4.4)	2 (9.1)	1 (3.1)	-5 (-22.7)	-7 (-28)		
October	1 (3.8)	2 (8.2)	-6 (-23.1)	-5 (-20.9)	-3 (-11.5)	-3 (-10.1)		
November	8 (30.8)	6 (22.5)	0	-2 (-7.5)	1 (3.8)	-1 (-5.1)		
December	-1 (-3.7)	-2 (-7)	1 (3.7)	0	-11 (-40.7)	-13 (-44.1)		
Total	-9 (-2.5)	-20 (-6.7)	87 (30.4)	72 (23.9)	-6 (-2.1)	-24 (-7.9)	0	-10 (-7.8)

There were a total 1048 and 1029.39 observed and expected deaths, respectively, resulting in an excess of 18.6 deaths (95% UI: 966.49 - 1092.29) using NBR analysis. Overall excess mortality and % excess mortality above expected levels using historical averages and NBR models were reported as 72 (7%) and 19 (1.8%), respectively (Figure 1).

Males had higher excess deaths (20) and % excess mortality (3.3%) as compared to females, who had lower than expected deaths (-3, -0.7%) (Table 2, Figure 3). Maximum excess mortality (%) was observed in adults aged 40-59 years (32; 43.8%), followed by 20-39 years (12; 40%), 60-79 years (20; 15.6%), and ≥80 years (6; 9.7%), and in the year 2021 as per the NBR model (Table 2, Figure 4). The highest overall excess deaths were observed in adults aged 40-59 years as per the historical average as well as the NBR analysis. Sensitivity analysis comparing trend vs. no-trend baseline models is shown in Figure 5. 

**Table 2:** Excess deaths through COVID-19 pandemic by gender, age, and year (January 2020-May 2023)

Month/Year	2020		2021		2022		Till May 2023	
	Excess deaths N (%)		Excess deaths N (%)		Excess deaths N (%)		Excess deaths N (%)	
	5-year average	Negative binomial regression	5-year average	Negative binomial regression	5-year average	Negative binomial regression	5-year average	Negative binomial regression
Gender								
Male	-9 (-5.2)	-13 (-7.4)	63 (36.6)	57 (32)	-11 (-6.4)	-19 (-10.6)	-1 (-1.4)	-5 (-6.6)
Female	1 (0.9)	-7 (-5.8)	25 (22.1)	15 (12.2)	6 (5.3)	-6 (-4.8)	2 (4.4)	-6 (-11.3)
Age								
<20	2 (20)	2 (20)	0	-1 (-9.1)	-5 (-50)	0	-1 (-33.3)	0
20-39	-2 (-7.4)	-5 (-16.7)	15 (55.6)	12 (40)	-7 (-26)	-7 (-26)	-1 (-11.1)	-5 (-38.5)
40-59	6 (9.4)	-2 (-2.8)	41 (64.1)	32 (43.8)	1 (1.6)	-9 (-12.2)	6 (20.7)	4 (13)
60-79	-17 (-13.4)	-17 (-13.4)	21 (16.5)	20 (15.6)	-3 (-2.4)	-6 (-4.6)	-4 (-7.7)	-6 (-11.1)
≥80	4 (7.1)	-2 (-3.2)	12 (21.4)	6 (9.7)	10 (17.9)	3 (4.8)	2 (8.7)	-2 (-7.4)

**Figure 3:**
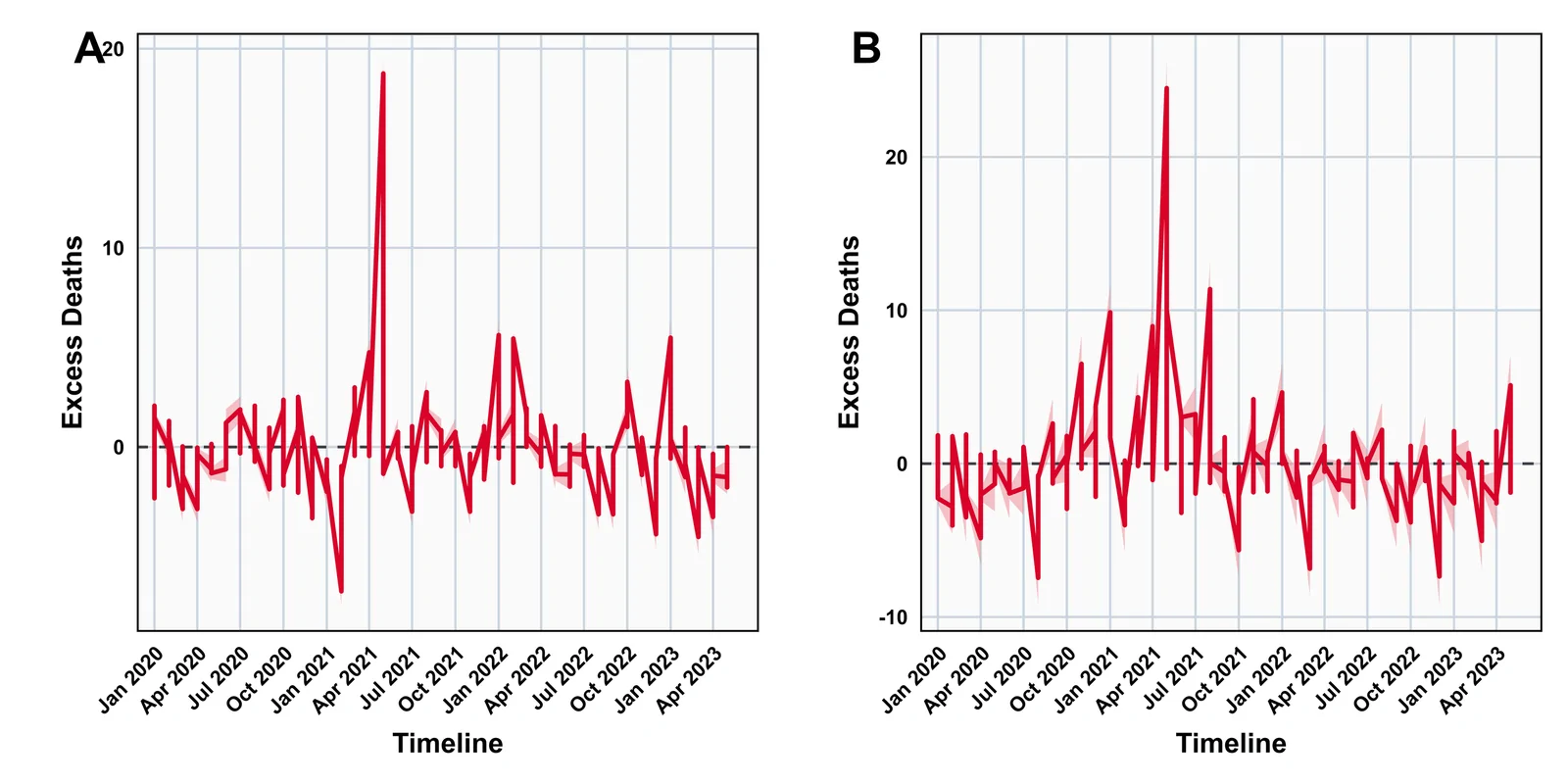
Gender-wise all-cause excess mortality (Jan 2020-May 2023) using the negative binomial regression model A) Female; B) Male

**Figure 4:**
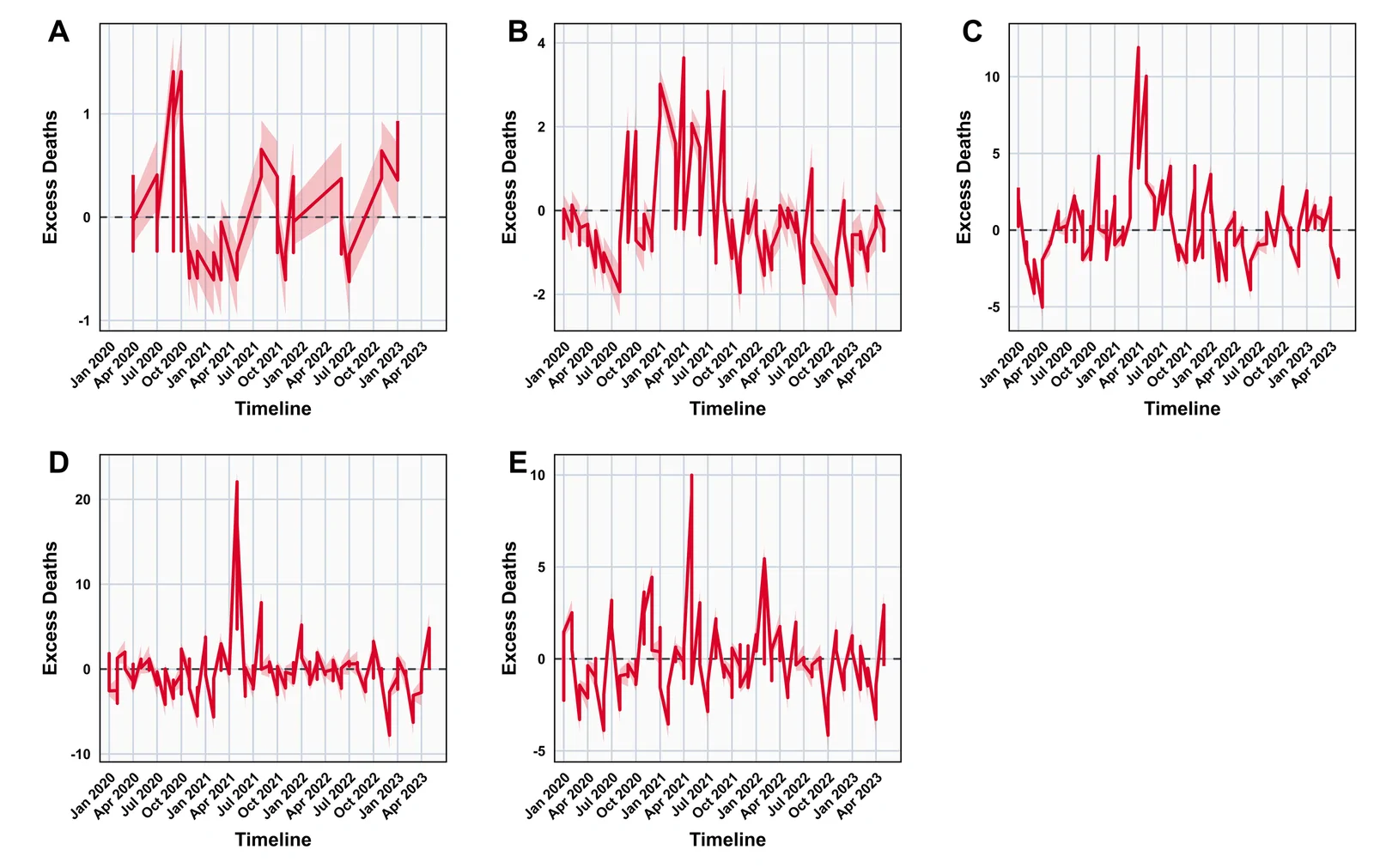
Age-wise all-cause excess mortality (Jan 2020 - May 2023) using the negative binomial regression model A) <20 years; B) 20-39 years; C) 40-59 years; D) 60-79 years; E) ≥80 years

**Figure 5:**
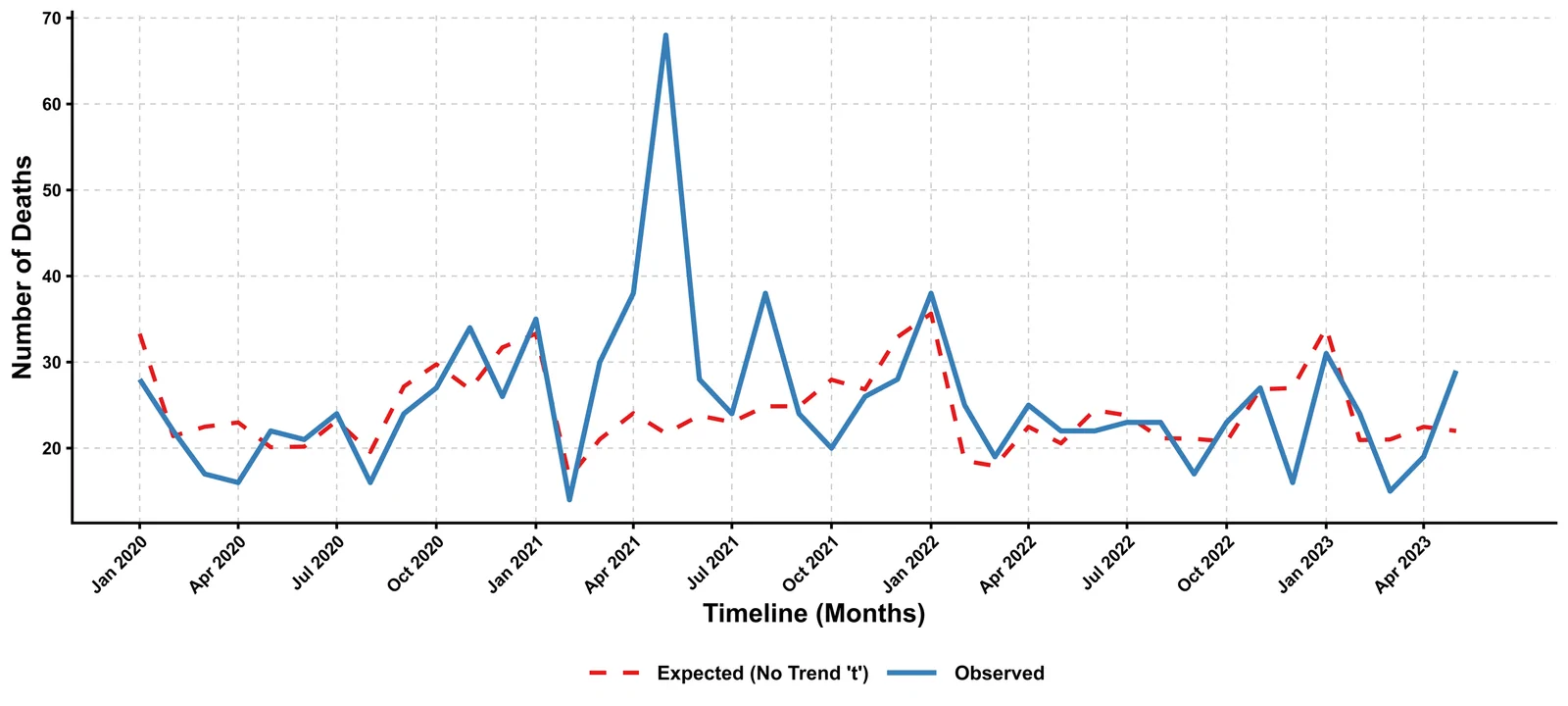
Monthly sll-cause mortality comparing trend vs. no-trend baseline models

## Discussion

The current study estimated all-cause mortality and excess deaths during the COVID-19 pandemic in a rural area of Punjab, India. It was observed that all-cause deaths surpassed the upper bounds (95% CI) of expected deaths in November 2020. There was negative excess mortality in the year 2020 followed by positive excess deaths in the year 2021. Months of April-June 2021 and August 2021 reported the highest excess mortality as per historical average and NBR models. Punjab recorded the first pandemic mortality on 18th March 2020 [12]. The first wave of COVID-19 was reported in 2020, with cases and deaths rising during mid-September. During this time of pandemic, the Government of India and Punjab made efforts to contain the widespread misinformation regarding disease transmission and vaccines, and implemented lockdown measures. The government and private sector worked in collaboration to provide quality COVID-19 management, including oxygen and hospital beds. This resulted in a fall in cases in January 2021. Driven by the spread of a new variant, as confirmed by whole-genome sequencing, as well as the lifting of restrictions, religious and political gatherings, cases and deaths started rising again in March 2021 and reached a peak in May 2021 during the second wave. Punjab even applied restrictions by implementing a night curfew to contain the disease [13,14].

In India, Punjab was probably the first state to officially report excess deaths based on historical averages (2016-2019) during the pandemic, and that analysis revealed over 52,000 excess fatalities corresponding to an 18.7% rise in mortality over the expected levels [8]. Three-quarters of the excess mortality was reported till June 2021 during the period of March-June 2021 [13]. Leffler et al., in an analysis of excess mortality in 18 Indian states, also revealed negative excess mortality in 2020 for six states, including Punjab, and a marked surge in all-cause mortality was observed across March to June 2021 in Punjab as well as Chennai, Kolkata, Delhi, Madhya Pradesh, Haryana, and Andhra Pradesh. Excess mortality of 120.3 per 100,000 population was recorded in Punjab by August 2021 [10].

Banaji et al. provided a comprehensive analysis of excess mortality using direct civil registration data in 12 states of India, including Punjab, and estimated 2.8-2.9 million excess deaths (28% from March 2020 to May 2021) at the national level using 2019 as the baseline year. An increase in excess deaths in June 2021 was also reported in Punjab, Karnataka and Andhra Pradesh (where data was available). Death registrations during this period were 123% higher than during June 2019 in these states. The ratio of excess deaths to recorded COVID‑19 deaths also increased from 8.0 in May 2021 to 11.7 in June 2021. However, it was stated that the surge in excess deaths in June, in their analysis, could be partly due to late registrations of May deaths [15]. Their findings underscore how severely the pandemic hit and how much of that impact was not captured in official reporting. 

Jha et al., in a comparison of COVID mortality in a telephone-based survey of a nationally representative sample with facility- and civil registration-based mortality data sources, revealed that COVID deaths accounted for 29% of all deaths between June 2020 and July 2021. Across approximately 200,000 public hospitals and smaller facilities (over 90% in rural areas), all‑cause deaths increased by 27% between July 2020 and 31st May, 2021. April-May 2021 alone accounted for a significant portion-0.45 million excess deaths (about 71% of the total), representing a 120% increase over the previous year. Increases in facility deaths were predominantly urban during the first wave, while the mortality surge spread to both urban and rural facilities in the second wave. The rise in all-cause mortality compared to the baseline period (2018-2019) varied substantially across states, with Gujarat and Punjab reporting a 230% and 87% surge in mortality, respectively [16].

An analysis by Malani et al. based on the Consumer Pyramids Household Survey (CPHS) reported that there were 4.2 million excess deaths through 2020 to Feb 2022 in India, with a second wave (delta) experiencing higher deaths (4.1 million) than the first wave of the original variant (2.2 million) [17]. Dedhe et al. in Pune, India reported 14,770 aggregate excess deaths during March 2020-Dec 2021 using a multi-method approach (simple average, Farington surveillance, overdispersed Poisson, death compensation claims, and wisdom of crowds) [18].

The increase in deaths reported in our study during 2021 was also in agreement with previous research conducted in India and other countries. Prior research by Acosta et al. [19] across Municipalities in Gujarat (44%), Lewnard et al. [20] in Chennai (41%) in India, Ruhm et al. [21] in United States (22%), Cilović-Lagarija et al. [22] in Bosnia and Herzegovina (32% in 2021, 18.2% from 2020-2023), Devanthal et al. [23] in Japan (3.7% during 2020-2023) and Ucar et al. [24] in Turkey (129%) also reported increase in mortality over the expected levels. Negative excess mortality during 2020 observed in our study was in line with various studies [21,24]; however, it was in contrast with other studies where positive excess mortality was observed during the first year of the pandemic [19,20,22].

Our study also revealed that from 2022 onwards, all-cause mortality returned to normal or baseline levels (negative excess mortality), as was also reflected by a decline in crude death rate to baseline levels after a sharp surge in 2021. Analysis by Malani et al. in India also reported negative excess mortality (-1.9 million) during the third wave (Omicron) of the pandemic [17]. Bosnia and Herzegovina also recorded a fall in excess mortality in 2022 [22]. However, Japan reported a surge in excess deaths in 2022 before experiencing a decline in 2023 [23]. India's third COVID‑19 wave, driven by the Omicron variant, was marked by rapid spread but had significantly lower severity. In addition, reduced mortality could possibly be due to effective implementation of containment measures, improved healthcare infrastructure coupled with widespread COVID-19 vaccination coverage. Punjab implemented containment through restrictions, scaling testing, the 'Corona Mukt Pind' campaign (in villages), spreading awareness, a state control room, oxygen management, and provision of more ICU beds. Punjab also implemented vaccination policy explicitly structured around geography, occupation, and demographic vulnerability [14]. A longitudinal analysis by Cheng et al. in 34 countries between 2020 and 2022 also reported a clear association between implementation of stronger containment measures and lower excess deaths during the periods of early as well as post-vaccine development [25]. Lewnard et al. reported a 7% decline in all-cause deaths following implementation of lockdown measures [26].

Since the end of the year 2023, the JN.1 variant of COVID-19 emerged as the dominant strain worldwide, which had characteristics of higher transmissibility because of some specific mutations in its spike protein [27]. In India also, the JN.1 variant emerged as the predominant SARS-CoV-2 variant, pressing the need for health authorities to predict and mitigate the risks effectively [28].

The present study reported higher excess mortality in males than females. This is in consonance with previous studies [19,20,21-24] that reported higher excess deaths among males across all age groups and during all the pandemic waves. Males had a greater risk of contracting COVID-19 infection and mortality due to their occupation and movement, especially after the lockdown was over. This was also reflected during an analysis by the International Institute for Population Sciences (IIPS), Deonar, India, where males had a 2.2-year drop in life expectancy as compared to 0.5 years among females during the pandemic, indicating that males died at higher rates than females [29]. Gender-related factors alone cannot adequately explain the observed disparities in excess mortality between females and males. Instead, they reflect the composite effects of gender wise social roles along with health-related behaviors, exposures to various occupations, and structural inequalities, especially those concerned with socioeconomic conditions [30].

Our study also revealed that adults aged 40-59 years and 80 years and above experienced higher excess mortality (by historical averages and NBR models), which is supported by observations of Cilović-Lagarija et al. (2025) in Bosnia [22]. Acosta et al. in Gujarat reported 33.7% and 34.1% excess mortality among adults aged 40-65 years and ≥65 years, respectively, and stressed the need for investigating the effect of social determinants and efficacy of public health policies on excess mortality [19]. In contrast, other studies in India [19,20] and outside India [21,23] reported varied increases in mortality across all age groups, with 20 years and above. As seen in the current study, other studies also documented that children below 15 years of age were not affected by excess mortality [19-22].

Both models (historical averages and NBR) estimated consistent trends in excess mortality. However, the NBR model estimated lower excess mortality than using historical averages, as it handles overdispersion and accounts for long-term trends and seasonal variations, whereas the historical averages model ignores population change and underestimates expected deaths if population size increases [3]. Therefore, it indicates that the estimated excess deaths depend on the type of method used in calculation, and one should always consider it while interpreting and comparing the results.

Strengths and limitations

The present study estimated excess mortality covering the entire duration of the COVID-19 pandemic period in a rural area of Punjab. In our study, every death was recorded by village-level health workers within the same time period/months as the deaths occurred. This, coupled with the absence of any other significant public health events at the time of the pandemic, indicates that the estimated excess mortality may largely be (directly or indirectly) attributed to the COVID-19 pandemic.

Our study has a few limitations too, as demographics, socioeconomic status, and vaccination status were not included, which might have provided more robust estimates of excess mortality. We could not estimate the undercount factor (excess deaths/official reported COVID-19 deaths) as separate official COVID-19 mortality data were not available for these 15 villages. A further limitation is the generalizability of the findings to other settings in Punjab and India. Demographic differences, socioeconomic status, implementation of various local government measures, health systems heterogeneity, and public response to the pandemic may limit its application in other settings. These limitations underscore the need for conducting continuous research and collecting data to enhance knowledge of excess mortality and its associated factors at regional and global levels.

## Conclusions

The study highlights all-cause excess mortality in rural Punjab. Although this rural area coped well during the first year of the pandemic with negative excess mortality, it gradually reversed, with increasing all-cause excess mortality, especially during the second year of the pandemic (2021), with April (59%) and May (167.4%) having higher excess mortality. The study witnessed high excess mortality across age groups, particularly among 40- 59-year-old adults and males. It highlights the importance of continuous surveillance of all-cause excess mortality and the need for more research to identify factors related to demographic and regional differences. Cause-specific analysis of deaths, coupled with data transparency and easy availability of data, may help in earlier implementation of pandemic containment strategies and strengthening health systems.
